# Cyanosis of Infants

**Published:** 1845-10

**Authors:** 


*Cyanosis of Infants.*—Dr. Meigs, Professor of Midwifery in Jefferson Medical College, read before the Academy of Sci-ences at its session June 16th, a note upon this subject. In-fants die in this case, said Dr. Meigs, from the presence of a black, veinous, non-oxygenated blood, in the encephalon; it is in the arteries of the brain that this blood becomes destructive to life, acting not as a poison, but simply because of its inca-pacity to excite the innervation in this organ. The whole world knows the anatomical cause of these phenomena—it is the persistance of the foramen ovale.

The occlusion of this foramen being prevented, because the sanguine torrent coming from the vena cava inferior, raises and keeps raised, the inter-auricular valve, which is thin and floating—Dr. Meigs conceived the idea of placing infants laboring under cyanosis upon the right side, with the head and trunk slightly elevated, in order that the inter-auricular septum might become horizontal, and that the blood contained in the left auricle might press with all its weight upon the valve which would thus be closed. Dr. Meigs has seen, that at the very instant when infants were placed in this position, the blue coloration would disappear, proving that there no longer penetrated into the arteries anything but oxygenated blood. Dr. Meigs affirmed that he had rescued from death from fifty to sixty infants in a hundred by this method, while all the other means employed to the present day, as is well known, have been unsuccessful.—*Gazette Médicale de Paris.*

